# For Better or for Worse? A Systematic Review of the Evidence on Social Media Use and Depression Among Lesbian, Gay, and Bisexual Minorities

**DOI:** 10.2196/10496

**Published:** 2018-07-23

**Authors:** César G Escobar-Viera, Darren L Whitfield, Charles B Wessel, Ariel Shensa, Jaime E Sidani, Andre L Brown, Cristian J Chandler, Beth L Hoffman, Michael P Marshal, Brian A Primack

**Affiliations:** ^1^ Center for Research on Media, Technology, and Health School of Medicine University of Pittburgh Pittsburgh, PA United States; ^2^ Center for LGBT Health Research University of Pittsburgh Pittsburgh, PA United States; ^3^ School of Social Work University of Pittsburgh Pittsburgh, PA United States; ^4^ Department of Psychiatry School of Medicine University of Pittsburgh Pittsburgh, PA United States; ^5^ Health Sciences Library University of Pittsburgh Pittsburgh, PA United States

**Keywords:** social media, social networking sites, sexual minorities, lesbian, gay, bisexual, depression, systematic review

## Abstract

**Background:**

Over 90% of adults in the United States have at least one social media account, and lesbian, gay, and bisexual (LGB) persons are more socially active on social media than heterosexuals. Rates of depression among LGB persons are between 1.5- and 2-fold higher than those among their heterosexual counterparts. Social media allows users to connect, interact, and express ideas, emotions, feelings, and thoughts. Thus, social media use might represent both a protective and a risk factor for depression among LGB persons. Studying the nature of the relationship between social media use and depression among LGB individuals is a necessary step to inform public health interventions for this population.

**Objective:**

The objective of this systematic review was to synthesize and critique the evidence on social media use and depression among LGB populations.

**Methods:**

We conducted a literature search for quantitative and qualitative studies published between January 2003 and June 2017 using 3 electronic databases. Articles were included if they were peer-reviewed, were in English, assessed social media use either quantitatively or qualitatively, measured depression, and focused on LGB populations. A minimum of two authors independently extracted data from each study using an a priori developed abstraction form. We assessed appropriate reporting of studies using the Strengthening the Reporting of Observational Studies in Epidemiology and the Consolidated Criteria for Reporting Qualitative Research for quantitative and qualitative studies, respectively.

**Results:**

We included 11 articles in the review; 9 studies were quantitative and cross-sectional and 2 were qualitative. Appropriate reporting of results varied greatly. Across quantitative studies, we found heterogeneity in how social media use was defined and measured. Cyberbullying was the most studied social media experience and was associated with depression and suicidality. Qualitative studies found that while social media provides a space to disclose minority experiences and share ways to cope and get support, constant surveillance of one’s social media profile can become a stressor, potentially leading to depression. In most studies, sexual minority participants were identified inconsistently.

**Conclusions:**

This review supports the need for research on the role of social media use on depression outcomes among LBG persons. Using social media may be both a protective and a risk factor for depression among LGB individuals. Support gained via social media may buffer the impact of geographic isolation and loneliness. Negative experiences such as cyberbullying and other patterns of use may be associated with depression. Future research would benefit from more consistent definitions of both social media use and study populations. Moreover, use of larger samples and accounting for patterns of use and individuals’ experiences on social media may help better understand the factors that impact LGB mental health disparities.

## Introduction

Despite growing acceptance and civil rights gains in recent years, lesbian, gay, and bisexual (LGB) individuals in the United States still face stigma and disparities regarding mental health conditions [1]. LGB persons are a diverse population whose sexual attraction, behavior, or orientation differs from their heterosexual counterparts. Importantly, estimated rates of depression among LGB persons are between 1.5- and 2-fold higher than their heterosexual counterparts [2]. In 2015, 14.8% of LGB males and 20.4% of LGB females in the United States suffered at least one major depressive episode, totaling 1.9 million individuals, compared with 4.3% of heterosexual males and 8% of heterosexual females [1]. These findings are consistent with those in other developed countries, and disparities are greater among bisexual adults [3,4].

Social media includes a variety of websites and mobile apps that enable users to create content and participate in online social networking (eg, YouTube, Tumblr, Facebook) [5]. It is estimated that well over 90% of adults in the United States have at least one social media account, with an average daily use of 2-4 hours [6,7]. Social media is a communication space where users express emotions, feelings, and thoughts. For LGB individuals, social media is a primary mode of socializing, and LGB persons are more socially active on social media than heterosexuals [8,9]. National data on LGB individuals found that over 85% of participants had one social media account, and they used it at least weekly; this usage rose to over 91% among LGB young adults [10].

Social media use encompasses a series of measures that capture the experience of using social media. Although no clear consensus exists regarding which specific measures should be counted as social media use, common ones include time (time elapsed while using social media over 24 hours), frequency (number of times people check their social media account per day) [11], number of friends and self-presentation [12], number of platforms (sites or apps) used [13], closeness to online friends, and activities performed (eg, posting updates, sharing pictures, etc) [14,15], as well as other use patterns (eg, active vs passive use, experiences such as experiencing cyberbullying, problematic use, positive or negative quality of interactions, and motivation to use social media) [16-22].

In a broader context, previous research has also investigated the linguistic attributes of social media use that indicate self-disclosure and mental health information sharing and that predict receipt of social support and other therapeutic outcomes on social media [23,24]. Moreover, several studies have looked at either the moderating or mediating role of upward social comparison [25-30], social connectedness [31], envy [32], and intensity of use [33].

Some of the complexities associated with social media use among LGB persons have been studied in relation with parenting and gender transition [34,35]. The findings of these studies point out potential stressors, such as the need to become incidental advocates or the task of detecting disapproval and allies within one’s social networks [34]. On the other hand, social media has some duality to it; despite being a stressful environment, it can also provide support that helps mitigate the said stress [35]. More specifically, while some researchers have found an association between social media use and increased risk of depression [11,13,16,36], others have found an association between specific patterns of use and improvement of psychological well-being [37]. Thus, social media use may be both a risk and a protective factor for depression and psychological well-being in the general population. In addition, using social media may add unique protective and risk factors for depression among LGB individuals.

Meyer’s minority stress theory is the predominant framework for understanding the protective and risk factors for depression and other mental health disparities among LGB individuals [38]. There are at least three fundamental tenets to the minority stress theory. First, exposure to LGB-related stressors such as discrimination, social rejection, and sometimes violent victimization is a central cause of mental health problems among LGB individuals. Second, exposure to these “distal” stressors is associated with “proximal” stressors such as internalized homonegativity and expectations of rejection. Third, social support from within the LGB communities can help offset or buffer the impact of these stressors on mental health outcomes. Meyer’s theory has also been extended to incorporate specific groups among LGB persons, such as children, adolescents, and people living with HIV [39,40]. While this theory helps explain the effects of social stress due to marginalized social identities on mental health outcomes among sexual minorities [38], the virtual social environment of social media introduces new complexities to previously described social interactions. For example, social media may make it easier for LGB individuals to disclose their sexual orientation to others by forming connections, providing education, and facilitating positive interactions and social support among LGB individuals. These virtual interactions may reduce the stress experienced by LGB individuals based on their sexual orientation and may protect sexual minorities from depression [41-43]. This may be particularly true for LGB individuals for whom it is too dangerous to be “out” or disclose their sexual minority identity in real-world settings such as in the workplace or in public social spaces. Conversely, social media use may be a vehicle for negative experiences, such as stigmatization and social comparison. These can lead to negative outcomes, including decreased self-esteem, and depressive symptoms [44,45].

The ubiquity of social media has led researchers to suggest its use to provide people with mental illness the opportunity to challenge stigma, provide and receive peer-to-peer support, and access either Web-based or mobile interventions [46]. To do this, we need to understand the mixed effects of social media use—improvement of psychological well-being or worsening of affective symptoms—on mental health. This understanding will inform policy and studies that leverage the positive aspects and address the potentially negative aspects of use as well. However, to date, no comprehensive synthesis of research on the impact of different patterns of use on depression among LGB populations has been conducted. Considering that LGB persons exhibit consistently high rates of social media use and consistently higher rates of depression than heterosexuals, this is a particularly important gap in the literature. For these reasons, we conducted a systematic review with four overarching goals: (1) identify all the peer-reviewed published papers that examined social media use and depression among LGB individuals; (2) describe the characteristics of these studies, including the study-appropriate reporting and methodology (eg, quantitative vs qualitative); (3) describe how social media use and depression constructs were operationalized across studies; and (4) assess which of the main tenets of the minority stress theory was analyzed in each study, in order to make recommendations for future studies that could leverage social media for improving depression outcomes in this population.

## Methods

### Inclusion and Exclusion Criteria

This systematic review has been reported in accordance with the Preferred Reporting Items for Systematic Reviews and Meta-Analyses Statement guidelines (Multimedia Appendix 1) [47,48]. The research protocol was registered in the PROSPERO database (#CRD42018088165) and is available as a supplement (Multimedia Appendix 2).

We included quantitative and qualitative studies published in peer-reviewed journals, in the English language, during or after 2003 (when MySpace, the first modern social media site, started operating). We allowed manuscripts from conference proceedings only when full-research papers were required for submission and each submission went through a complete peer-review process. Included studies had to focus on social media use and depression among LGB minorities. We defined social media use as any usage measurement (eg, time, frequency, motivation to use, experiences while using, etc). Depression comprised major depressive disorder, bipolar depression, dysthymia, depressive symptoms, and psychological distress. LGB minorities were defined as lesbian, gay, bisexual, and men who have sex with men. Exclusion criteria included theses or dissertations, opinion pieces or reviews, and articles that studied use of short message service text messages (not included in our definition of social media). Research in which the sole focus was on gender minority populations (eg, transgender and gender nonbinary) were excluded to avoid conflating results of sexual minorities, which may not be applicable to gender minorities. However, studies in which gender minorities were a subpopulation included in the study LGB sample were included.

### Search Process

Literature searches were developed and executed by a health sciences librarian (CBW) in PubMed or MEDLINE (1946-Present), PsycINFO, Ovid (1806-present), and SocINDEX, EBSCOhost (1895-present). Controlled vocabulary from Medical Subject Headings (MeSH), the Thesaurus of Psychological Index Terms, and SocINDEX Subject Terms, along with keywords and descriptors were used for the concepts of lesbian, gay, bisexual, transgender, queer, and interse (LGBTQI), social media, and depression. For all three concepts, we included MeSH and text words in title and abstract. For social media, descriptors included sexual network or partner; seeking sex on internet, online, on the Web, or on websites; finding partners on the internet, online, on the Web, or on websites; and sexual behavior on the internet, online, on the Web, or on websites. For depression, descriptors also included all related depression terms. The Boolean operator “AND” combined the three search components. Searches were limited to journal articles only, with no language or publication year restrictions. The entire list of keywords, descriptors, and search strings used in each database is available as a supplement (Multimedia Appendix 3). Search results were downloaded and imported into an EndNote Library on June 5, 2017. A total of 1259 citations were found. Of these, 539 citations were from PubMed or MEDLINE, 404 citations from PsychINFO, and 316 citations from SocINDEX. There were 160 duplicates records, leaving 1099 citations to screen.

### Study Selection and Data Extraction

Screening and data extraction were completed using DistillerSR [49]. Structured forms were uploaded to the software and used throughout the entire process. Six researchers (ALB, CJC, BLH, AS, JES, and DLW) independently screened all article titles and abstracts to generate a set of references for which there was any possibility for selection. Next, these six researchers were divided into three pairs and were randomly assigned an equal number of references; they assessed the full text of these studies to determine eligibility. Interrater reliability was substantial (weighted Cohen kappa, 0.70) [50]. To minimize the risk of reviewer bias, consensus meetings between the first author and each pair of reviewers to resolve differences occurred, but only after independent screening of all articles. In one case, the first author adjudicated a reference for inclusion.

Extraction forms included seven categories of information: (1) study logistics (setting, country, publication year, social media site under study, study design, and funding source); (2) study population characteristics (number of subjects, age, gender, race or ethnicity, sexual minorities included, education level, and income); (3) social media use (number of social networking sites, time of usage and frequency, scales, and contextual measures); (4) health outcomes measured (primary and secondary outcomes measured and scales); (5) main results and limitations; (6) main tenet of the minority stress theory under study (ie, distal stressors, proximal stressors, and social support); and (7) appropriateness of reporting. To ensure accuracy, we implemented a quality-control mechanism in which one reviewer completed a first data extraction and the second reviewer validated or disagreed with it. Again, disagreements were resolved in consensus meetings with each pair and the first author.

### Appropriate Study Reporting

We assessed the appropriate reporting of the included studies. For quantitative studies, we used the Strengthening the Reporting of Observational Studies in Epidemiology (STROBE) Statement Checklist v4.0 [51,52]. The STROBE statement consists of a checklist of 22 items related to all sections of research manuscripts; STROBE provides reporting recommendations for studies that investigate associations between exposures and health outcomes [51,52]. We assigned values of 0-1 to each check mark. Thus, total score for each manuscript could range from 0 to 22, in which 22 means the study fully met the STROBE standards of appropriate reporting. For qualitative manuscripts, we used the Consolidated Criteria for Reporting Qualitative Research (COREQ-32) [53], a checklist of 32 items aimed at improving the quality of reporting individual interviews– and focus groups–generated data. We used the same previously explained mechanism to score each manuscript from 0 to 32, in which 32 means the study fully met the COREQ-32 standards of appropriate reporting. Each study was appraised by at least two reviewers, and the first author was consulted to resolve any disagreement. The assessments of appropriate reporting for all manuscripts are available as supplements (Multimedia Appendices 4 and 5).

## Results

### Study Identification

**Figure 1:**
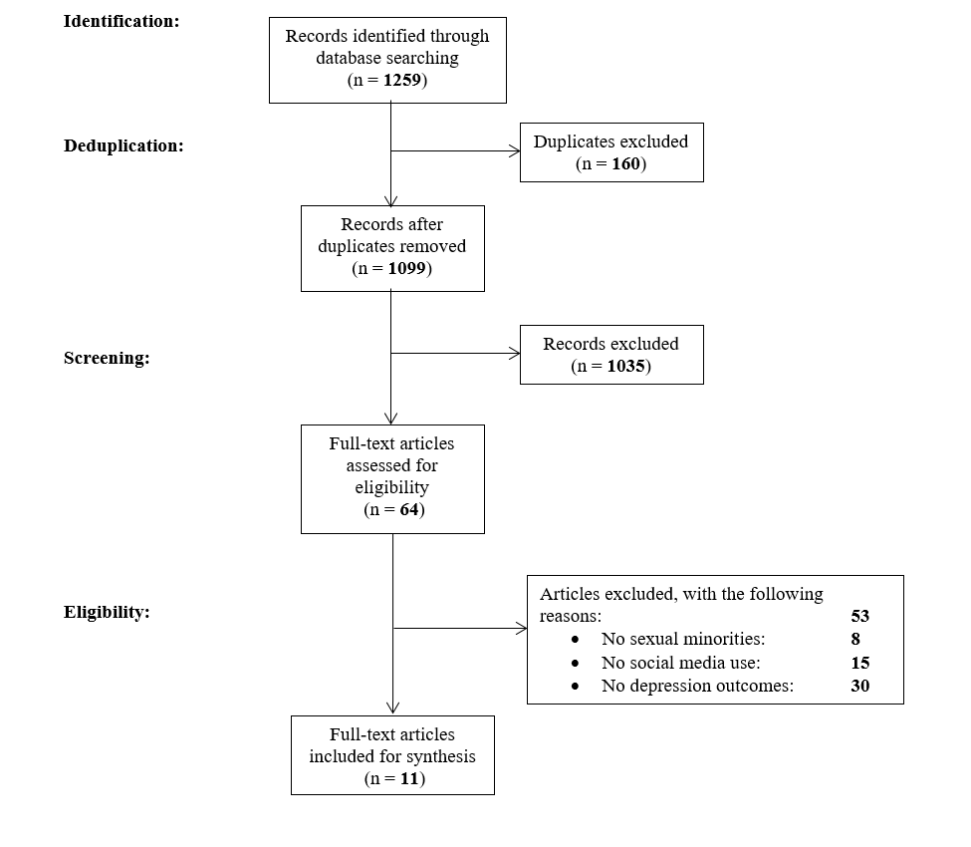
Flowchart of studies screened and included in a 2017 systematic review of social media use and depression among lesbian, gay, and bisexual minority populations.

We identified 1259 records through our database searching process. After excluding duplicates, we reviewed 1099 unduplicated citations (Figure 1).

Of these, 1035 were excluded after title and abstract screening. Of the 64 full-text manuscripts that were assessed for eligibility, 53 were excluded for different reasons: 8 lacked a focus on sexual minorities, 15 did not specifically assess social media use, and 30 did not have depression as part of the outcomes under study. Eleven research articles were, thus, included in the final sample. Reference lists of included articles were examined for additional studies. However, no new study that met the inclusion criteria was identified using this method.

### Study Characteristics and Appropriate Reporting

Of the 11 included studies, 8 (72%) consisted of cross-sectional surveys [54-61], 2 (18%) consisted of qualitative analyses [62,63], and 1 (9%) combined cross-sectional surveys with social network analysis [64] (condensed Table 1; for full table, please see Multimedia Appendix 6). The social media site or platform targeted in the study varied across the included manuscripts. Of the 11 studies, 5 (46%) did not focus on a specific social media site [54,56,57,60,61], 2 (18%) targeted the use of blogs and discussion forums [59,63], and 4 (36%) focused on a specific social media site or platform [55,58,62,64].

**Table 1:** Characteristics of studies on social media use and depression including sexual minorities published between January 2003 and June 2017.

Author(s), country, year	Design	Social media site or app	Participants			Score^a^
			N	Sample description	Sexual minorities (%)	
Morelli et al, Italy, 2016 [54]	Cross-sectional survey	No specific site	1334	Middle- and high-school students and young adults	Lesbian or gay (12.6)	11^b^
Gibbs & Rice, USA, 2016 [55]	Cross-sectional survey	Grindr	195	Male users of a hook-up mobile app	Gay (86); bisexual (9.8)	20^b^
Cenat et al, Canada, 2015 [56]	Cross-sectional survey	No specific site	6540	Students from 34 participating high schools across Canada	Lesbian or gay (1.3); bisexual (10)	20^b^
Rubin & McClelland, USA, 2015 [62]	Individual interviews	Facebook	8	Female adolescent who reported being daily Facebook users	Lesbian (62.5); bisexual (37.5)	15^c^
Duong & Bradshaw, USA, 2014 [57]	Cross-sectional survey	No specific site	951	Sexual minority students, grades 9-12 from 105 NYC^d^ schools	N/A^e^	17^b^
Homan et al, USA, 2014 [64]	Cross-sectional survey; social network analysis	TrevorSpace	195	Users of a LGBQ^f^ social networking site	N/A	19^b^
Lester, USA, 2006 [58]	Cross-sectional survey	Bmezine	4700	Users of a body modification website	Lesbian or gay (5); bisexual (37.9)	5^b^
Cooper & Blumenfeld, USA, 2012 [59]	Cross-sectional survey	General blogs and discussion boards	310	National sample of middle- and high-school students who identified as LGB^g^, or with same-sex attraction or LGBT^h^ allied youth	Lesbian or gay or bisexual (80.6)	13^b^
Alang & Fomotar, USA, 2014 [63]	Netnography	Unidentified forum for new and expecting parents	N/A	Messages from an unidentified online forum for lesbian mothers with postpartum depression	Lesbian (100)	17^c^
Ceglarek & Ward, USA, 2016 [60]	Cross-sectional survey	No specific site	570	College students and community LGBTQ^i^ group members	Lesbian or gay (6.8); bisexual (5.4); predominantly heterosexual (13.5)	20^b^
Ramsey et al, USA, 2016 [61]	Cross-sectional survey	No specific site	634	Undergraduate college students	Lesbian or gay (7.6); bisexual (4.4); mostly gay or lesbian (3.5); mostly heterosexual (3.3); questioning (0.8)	19^b^

^a^Appropriate reporting score. Reporting adequacy was assessed using footnotes b and c.

^b^The Strengthening the Reporting of Observational Studies in Epidemiology (range 0-22) for quantitative studies.

^c^The Consolidated Criteria for Reporting Qualitative Research 32 (range 0-32) for qualitative studies.

^d^NYC: New York City.

^e^N/A: not applicable.

^f^LGBQ: lesbian, gay, bisexual, and questioning.

^g^LGB: lesbian, gay, and bisexual.

^h^LGBT: lesbian, gay, bisexual, and transgender.

^i^LGBTQ: lesbian, gay, bisexual, transgender, and questioning.

The studies that captured the age of participants [54-56,59-62,64] reported that ages ranged between 11 and 30 years; 27% (3/11) studies did not report participants’ age range [57,58,63]. Furthermore, 64% (7/11) studies included both male and female participants [54,56-61]; in these studies, female participants ranged from 55% to 78%. In addition, 18% studies (2/11) included only female participants [62,63] and 9% (1/11) had an exclusively male sample [55]; 18% (2/11) studies included a sample of transgender participants, with this group comprising 0.9% [59] and 0.8% of participants [61]. Additionally, 18% (2/11) studies included a small sample of gender nonconforming participants. In these studies, the percentage of gender nonconforming participants ranged from 0.7% [58] to 1.6% [61]. Participants’ gender was not reported in 9% (1/11) studies [64].

Overall, the reporting of sexual minority participants varied across studies; 46% (5/11) of studies combined gay and lesbian identity [54,56,58,60,61]; 18% (2/11) studies reported a “predominantly or mostly heterosexual identity” category [60,61] and 9% (1/11) combined gay, lesbian, and bisexual identities [59]. Furthermore, 18% (2/11) studies reported that their entire sample comprised LGB participants, but participants’ sexual orientation was not broken down into specific categories [57,64].

Finally, the appropriate reporting of results was variable. Among 9 quantitative studies, STROBE scores [51] ranged from 5 to 20 out of 22. Of all, 89% (8/9) studies met the reporting standards on their title, abstract, and introduction sections; 67% (6/9) studies met reporting standards on methods, 22% (2/9) on results, 6 (67%) on discussion, and 2 (22%) on funding source reporting (Multimedia Appendix 4). For 2 qualitative studies, COREQ-32 scores [53] were 15 [62] and 17 [63] out of 32, respectively (Multimedia Appendix 5).

### Exposure and Outcome Characteristics and Social Media Sites Studied

Operationalizing of social media use measurement varied across studies, and these findings are summarized in a condensed Table 2 (for full table, please see Multimedia Appendix 7). In 9 quantitative studies, social media use was assessed in variable ways: 33.3% (3/9) studies measured the self-reported experience of cyberbullying [56,57,61], 22.2% (2/9) measured the frequency of social media use [59,60], and 22.2% (2/9) assessed the general use of social media (dichotomously) [55,58]. Furthermore, 44.4% (4/9) studies measured only one of the following: sexting behavior [54], integration of social ties on social media [64], number of social media platforms used [60], and motivation to use social media [60]. Qualitative studies explored the use of Facebook profile management tools [62] and experience of using an online support forum [63].

Similarly, assessment of depression varied across quantitative studies. Depression was operationalized as depressive symptoms in 44.4% (4/9) [55,59-61,64], psychological distress in 22.2% (2/9) [54,56], and suicidality in 33.3% (3/9) [56-58] studies; 22.2% (2/9) studies assessed either engagement in physical fights [57] or emotional responses to cyberbullying (including feelings of depression) [59]. Qualitative studies analyzed depression-related themes including social, emotional, and health consequences of stress caused by managing participants’ Facebook profiles [62] and the emotional experience derived from using an online support forum [63].

### Main Findings

Exposure to cyberbullying on social media among LGB individuals was frequent, and the majority of those who experienced it reported feelings of depression [59] (condensed Table 2; for full table, please see Multimedia Appendix 7). Compared with heterosexual youth, bisexual boys and girls were more likely to report cyberbullying [56,61]. Among LGB boys and girls, cyberbullying was directly and independently associated with psychological distress [56], depression [61], engaging in physical fights [57], and suicidal thoughts or suicide attempts [56,57]. Compared with heterosexuals, sexual minority users of an online forum group also had higher rates of suicidality [58].

Association between using social media and depression differed depending on which characteristic or pattern of use was under study. One study found that lesbian or gay participants had higher rates of sexting behavior than their heterosexual peers; however, psychological distress was not different across three levels of sexting [54]. Another study found moderate levels of depression among all male users of a gay hook-up mobile app [55]. Yet another study found that social media users who had more friends who knew each other (ie, a tightly integrated social network) predicted lower depression scores than those who did not [64]. When compared with their heterosexual peers, sexual minority youth reported higher rates of both social media sites used and motives to use them [60]. Furthermore, perceived social support on social media among sexual minority youth was negatively associated with loneliness and using social media to discuss LGB issues was negatively associated with anxiety and hostility [60].

Qualitative explorations about social media use and mental health among sexual minorities indicated both risks and benefits. Maintaining a Facebook profile was deemed part of everyday life among lesbian and bisexual females [62]. However, it also requires constant surveillance and monitoring of one’s social interactions, which in turn can be a stressor, leading to rumination of ideas, shame, and depression if one is excluded or outed [62]. On the other hand, among LGB mothers dealing with postpartum depression, an online forum served as a space where they could disclose their experiences with the condition while sharing ways to cope with it, building a community that provided different forms of social support [63].

Finally, while most of the reviewed studies assessed only one component of Meyer’s theory, other studies focused on more than one. Of the included studies, 81.8% (9/11) focused on social media experiences as a source of stressors, such as victimization or cyberbullying [54-59,61,62,64]; 36.4% (4/11) studies focused on social media as a potential source of support for LGB individuals [55,60,63,64] and 9.1% (1/11) assessed proximal stressors (ie, sexual orientation identity concealment on social media) [62].

**Table 2:** Exposure and outcome assessment and main findings of studies of social media use and depression including sexual minorities published between January 2003 and June 2017.

Author(s), country, year	Exposure assessment tool	Outcome assessment tool	Aspect of minority stress theory studied
Morelli et al, Italy, 2016 [54]	Modified version of the Sexting Behaviors Scale	12-item General Health Questionnaire	Distal stressors
Gibbs & Rice, USA, 2016 [55]	Sample was recruited exclusively from Grindr (overall use was not assessed)	4-item Center for Epidemiological Studies Depression Scale	Distal stressors; social support
Cenat et al, Canada, 2015 [56]	Item asking, “In the last 12 months, how many times someone has bullied you (rumors, intimidation, threatening, etc) using the internet (Facebook, MySpace, MSN, email, text, etc)?”	10-item Kessler Psychological Distress Scale; item asking, “Have you ever seriously thought of committing suicide?”	Distal stressors
Rubin & McClelland, USA, 2015 [62]	Experience of being young, queer, and a person of color in an online network	Consequences of social exclusionary practices within an online network	Distal stressors; proximal stressors
Duong & Bradshaw, USA, 2014 [57]	Item from Youth Risk Behavior Survey asking, “During the past 12 months, have you ever been electronically bullied, such as through email, chat rooms, instant messaging, websites, or text messaging?”; item asking, “In the past 12 months, have you ever been bullied on school property?”	Item asking, “During the past 12 months, how many times did you actually attempt suicide?”; “During the past 12 months, how many times were you in a physical fight?”	Distal stressors
Homan et al, USA, 2014 [64]	Social network structure graph	9-item Patient Health care Questionnaire	Distal stressors; social support
Lester, USA, 2006 [58]	Sample was recruited exclusively from Bmezine (overall use was not assessed)	Item asking, “How many times have you attempted suicide?”	Distal stressors
Cooper & Blumenfeld, USA, 2012 [59]	Item asking, “How often in an average week do you use communication technologies (eg, blogging, chat rooms, and discussion boards)?”; item asking, “How often in the last 30 days have you been harassed based on your sexual identity?”	Not provided	Distal stressors
Alang & Fomotar, USA, 2014 [63]	Assessment of the role of an online forum as source of social support	Experience of lesbian mothers with postpartum depression using a dedicated online forum	Social support
Ceglarek & Ward, USA, 2016 [60]	Item asking, “How often do you use social networking sites?”; “Which social networking sites do you use?”; “How much these statements apply to you?” Example statement: “I use social networking sites to seek groups of people similar to myself”	26-item Brief Symptom Inventory	Social support
Ramsey et al, USA, 2016 [61]	Cyberbullying; Victimization Scale of the Cyberbullying and Online Aggression Survey	Center for Epidemiological Studies Depression Scale-Revised	Distal stressors

## Discussion

### Principal Results

In this systematic review, we found a low number of peer-reviewed published research examining social media use and depression among LGB persons. We found ample variation in measurement of social media use and operationalization of these measures. Variability across studies was also found in the definition of sexual minorities as well as conflating sexual and gender minorities in the same study population. The implications of these findings and suggestions for future research are discussed below.

Despite our comprehensive inclusion criteria and systematic online search approach (eg, we included articles that measured depressive symptoms using a psychologic distress scale), there were few studies that examined the relationship between social media use and depression among LGB individuals; 9 studies were cross-sectional and only 2 examined qualitative data. Appropriate reporting of results was variable across the included studies. For quantitative studies, most of the variability was due to incomplete reporting of study results, such as demographics, clinical and social characteristics of participants and reasons for nonparticipation, incomplete report of estimates, and nonreporting of ad-hoc analyses (eg, interactions, sensitivity analysis). On the other hand, most of the variation across the 2 qualitative studies was due to inadequate reporting of sample size, nonparticipant characteristics, sample description, development of interview guides, or data saturation.

We found variation in the included studies’ approach to whether assess social media use on a platform-specific approach or for social media as a whole. While social media sites share commonalities, in many aspects they are also very different. For example, certain social media sites are more popular among certain groups than others [65]. Moreover, while some actions and modes of interaction (eg, posting a picture; live streaming; and commenting on someone’s tweet, post, or status update) are actions one can perform across several social media sites, the length of time the picture or video is available, the allowed length of response to a comment, and the audience for these can be very different from one social media site to another. It is also important to consider the motivation to use a given site.

A key finding from this review, adding to other research on the same topic [36,66], is that seeking social support and connectedness might be a potentially strong motivation to use social media among LGB individuals [60,62,63]. Social support is a known protective factor against depression [67-69]. This review supports the need for future research that focuses on assessing the role of variables that describe the quality of the social media experience (eg, active and passive use, motivation to use) in order to understand the effect of social media use on depression among LGB persons.

There was considerable variation in how social media use was operationalized and measured among the included studies, which speaks to the complexity of using social media. In some studies, use was operationalized in terms of frequency, the number of platforms used, and for how long the individuals used them. In other studies, use was measured in terms of characteristics, such as experiences with cyberbullying and use of social media to find camaraderie online. These findings are consistent with those of other systematic reviews linking social media use to mental health outcomes within the general population [36,66]. For example, while some studies have found frequency or volume of social media use to be associated with depression [11,70], these studies do not take into consideration the specific activities undertaken on social media (eg, engaging in contentious interactions or comparing one’s self to others) that could be associated with depression. It is possible that behaviors such as scrolling through newsfeeds with little interaction could also be a problematic behavior. Any of these actions—which vary greatly but may all yield differing levels of importance to mental health outcomes—may be categorized as social media use. While studies using various measures of social media use add to the richness and understanding of it, they may lead to false comparisons and mixed results. It may be valuable for future research to conduct scale development studies that focus on social media use as a construct. Additionally, use of clear and transparent language that more accurately defines the measurements of use may be beneficial.

The results of this review echo a body of research that found an elevated prevalence of depression and psychological distress among LGB individuals compared with that among their heterosexual counterparts [71-75]. Our findings point to the variability in experiencing depression and psychological distress in association with social media use for bisexually identified individuals. These findings might be explained by the minority stress theory [38], which posits that individuals with marginalized identities experience stress from their social environment due to social status [76]. The higher rates of depression and psychological distress among LGB persons may be attributed in part to experiencing discrimination, harassment, and victimization because of their sexual orientation. The findings that using social media may be a protective factor articulates the argument of the sense of an LGB community, which suggests that belonging to a larger community may buffer the effects of marginalization [77,78]. In terms of social media, being connected to other LGB individuals may reduce the psychological effects of discrimination, harassment, and victimization these persons experience in the social environment. Nevertheless, the small samples of LGB individuals in these studies limit the ability to determine if any subgroup differences exist in the protective nature of community connectedness.

This review found a strong focus within the literature on environmental and societal stressors that, via social media, may impact depression outcomes among LGB persons. Much less emphasis has been put on understanding the role of online social support and proximal stress processes (eg, expectation of rejection, concealment, and internalized homophobia) in the association between social media use and depression in this group. In addition, it is not clear to what extent experiences lived in the offline world translate to the social media world for LGB persons. For example, while social support is an important protective factor for depression, the findings regarding online social support in the general population have been mixed, with one study reporting lower levels of protection against adverse mental health outcomes [67]and another reporting an improved quality of life and well-being among those who feel socially excluded and seek online social support [79]. Future studies should keep expanding research on the different components of the minority stress theory as they relate to social media use and depression.

Importantly, we found methodological concerns across the included studies regarding the definition of sexual minority individuals, as well as the conflation of results from men and women in the sample, making it difficult to interpret as to which group the said results would apply. We found a lack of clearly defined LGB samples in this area of research. Of the 11 included studies, 2 did not report participants’ sexual orientation. Among studies that reported sexual orientation, several grouped gay men and lesbian women into one group. This reduction limits the ability to understand how using social media may be associated with psychological distress and depression in each group separately. Upon further analysis, studies that included both sexual orientation and gender identity often conflated these two groups, making it hard to determine the differences in experiences of depression based on sexual orientation or gender identity. The collapsing of groups complicated our ability to understand the nuanced differences experienced by individuals based on sexual orientation and gender identity [80]. These findings suggest the need for research that includes larger samples of LBG participants to allow the study of lesbian women, gay men, bisexual women, and bisexual men separately, as well as samples that allow distinguishing sexual orientation from gender identity when reporting results. The lack of representativeness extends to other subpopulation differences. For example, half of the studies did not report participant race or ethnicity, and among those that did, the racial composition of the samples was predominantly white. However, research suggests that LGB racial or ethnic minorities have different experiences with both social media use and mental health compared with their white counterparts [65,81,82]. Thus, considerations should be made to ensure these samples include adequate percentages of LGB racial or ethnic minorities.

None of the studies included in this review had participants older than 30 years of age. While young adults are the group with highest levels of social media use, around 70% of adults aged 30-64 years and over 35% of those aged 65 years and above have at least one social media account [65]. Given the higher risk of depression among the LGB population and the potential dual role of social media, the lack of data from older individuals is concerning. Usage of, interaction with, and experiences concerning social media may be different by age group, and these variations could have differential effects on mental health outcomes. Future research focused on improving the sampling of sexual minority populations should also consider improving sampling across different age groups.

### Implications and Future Directions

Internal and social stressors related to minority status are at the core of the minority stress theory [38]. Given the global spread of social media as both a tool and environment within which social interactions occur, we may need a potential expansion of Meyer’s theory, one that accounts for the social media experience. Elements of this theory can be applied to LGB-related experiences in the social media environment. However, Meyer’s theory was published in 2003, at a time when many of the modern social media sites that are used today did not exist. For example, MySpace started in the same year that Meyer’s work was published, and Facebook started the following year; since then, there has been a proliferation of various social media sites and platforms, which have dramatically changed the social interaction landscape of LGB individuals. As social media research progresses, we need to empirically test the relationship between the different components of Meyer’s theory, social media use, and depression. This will inform whether social media aggravates or alleviates minority stress and depression, as well as how and to what extent. Moreover, we expect that as the field moves forward, this research could potentially reveal new or modified risk and protective factors for LGB individuals’ mental health in ways the minority stress theory could not anticipate. Understanding how virtual and nonvirtual social platforms influence mental health, both independently of and interacting with each other, will be critical to gain a full understanding of the role of the social environment on LGB mental health disparities.

### Conclusions

There is a growing concern about the impact of social media use on mental health outcomes. LGB individuals are a well-suited population to study the nature of the relationship between social media use and depression due to the disproportionately high prevalence of both in this group. This systematic review supports the need for research that addresses the role of using social media in the pathway of depression and other mental health outcomes among sexual minority populations. Our findings suggest that social media use may be both a protective and risk factor for these outcomes among LGB individuals. Connections and support gained via social media may buffer the impact of geographic isolation, discrimination, and loneliness that some LGB persons experience in their daily lives. However, the pressure of maintaining a desirable social media presence, negative experiences on social media such as cyberbullying, and certain patterns of use may associate with increased depressive symptoms in this population. Our findings also indicate the need for future research in this field to recruit larger samples, have more consistent definitions of the study populations, better define the social media use construct, and incorporate the social media experience into the conceptualization of psychosocial factors that impact sexual minorities’ mental health disparities.

## Appendix

Multimedia Appendix 1 Preferred Reporting Items for Systematic Reviews and Meta-Analyses (PRISMA) checklist.

Multimedia Appendix 2 PROSPERO research protocol.

Multimedia Appendix 3 Online search strategy.

Multimedia Appendix 4 Evaluation of quantitative studies included using the Strengthening the Reporting of Observational Studies in Epidemiology (STROBE) criteria.

Multimedia Appendix 5 Evaluation of qualitative studies included using the Consolidated Criteria for Reporting Qualitative Research (COREQ-32).

Multimedia Appendix 6 Characteristics of studies of social media use and depression including sexual minorities published between January 2003 and June 2017.

Multimedia Appendix 7 Exposure and outcome assessment and main findings of studies of social media use and depression including sexual minorities published between January 2003 and June 2017.

## Abbreviations

COREQ-32: Consolidated Criteria for Reporting Qualitative Research
LGB: lesbian, gay, and bisexual
LGBQ: lesbian, gay, bisexual, and questioning
LGBT: lesbian, gay, bisexual, and transgender
LGBTQ: lesbian, gay, bisexual, transgender, and questioning
LGBTQI: lesbian, gay, bisexual, transgender, queer, and intersex
MeSH: Medical Subject Headings
STROBE: Strengthening the Reporting of Observational Studies in Epidemiology
