# Supplementary material for: For Better or for Worse? A Systematic Review of the Evidence on Social Media Use and Depression Among Lesbian, Gay, and Bisexual Minorities
Source: JMIR Ment Health. 2018 Jul 23;5(3):e10496. doi: 10.2196/10496 (PMC6079300; doi:10.2196/10496)
Supplement: Multimedia Appendix 2 [file mental_v5i3e10496_app2.pdf]

PROSPERO
International prospective register of systematic reviews

National Institute for Health Research

Systematic review

1. * Review title.

Give the working title of the review, for example the one used for obtaining funding. Ideally the title should state succinctly the interventions or exposures being reviewed and the associated health or social problems. Where appropriate, the title should use the PI(E)COS structure to contain information on the Participants, Intervention (or Exposure) and Comparison groups, the Outcomes to be measured and Study designs to be included.

Evidence on social media use and depression among sexual minorities: a systematic review

2. Original language title.

For reviews in languages other than English, this field should be used to enter the title in the language of the review. This will be displayed together with the English language title.

3. * Anticipated or actual start date.

Give the date when the systematic review commenced, or is expected to commence.

01/06/2017

4. * Anticipated completion date.

Give the date by which the review is expected to be completed.

28/02/2018

5. * Stage of review at time of this submission.

Indicate the stage of progress of the review by ticking the relevant Started and Completed boxes. Additional information may be added in the free text box provided.
Please note: Reviews that have progressed beyond the point of completing data extraction at the time of initial registration are not eligible for inclusion in PROSPERO. Should evidence of incorrect status and/or completion date being supplied at the time of submission come to light, the content of the PROSPERO record will be removed leaving only the title and named contact details and a statement that inaccuracies in the stage of the review date had been identified.
This field should be updated when any amendments are made to a published record and on completion and publication of the review.

The review has not yet started: No

**Review stage**	**Started**	**Completed**
Preliminary searches	Yes	Yes
Piloting of the study selection process	Yes	Yes
Formal screening of search results against eligibility criteria	Yes	Yes
Data extraction	No	No
Risk of bias (quality) assessment	No	No
Data analysis	No	No

Provide any other relevant information about the stage of the review here (e.g. Funded proposal, protocol not yet finalised).
We expect to finalize the coding and summary of findings within a month. We ended up with only 11 studies eligible for inclusion in the review. We have 3 pairs of reviewers using an online software for systematic reviews to help streamline the entire process. Therefore, we believe we will complete the review within the stated time frame.

We expect to finalize the coding and summary of findings within a month. We ended up with only 11 studies eligible for inclusion in the review. We have 3 pairs of reviewers using an online software for systematic reviews to help streamline the entire process. Therefore, we believe we will complete the review within the stated time frame.

6. * Named contact.

The named contact acts as the guarantor for the accuracy of the information presented in the register record.

César G. Escobar-Viera

Email salutation (e.g. "Dr Smith" or "Joanne") for correspondence:

Dr. Escobar-Viera

7. * Named contact email.

Give the electronic mail address of the named contact.

escobar-viera@pitt.edu

8. Named contact address

Give the full postal address for the named contact.

Suite 600
230 McKee Place
Pittsburgh, PA 15213, USA

9. Named contact phone number.

Give the telephone number for the named contact, including international dialling code.

(412) 692-4297

10. * Organisational affiliation of the review.

Full title of the organisational affiliations for this review and website address if available. This field may be completed as 'None' if the review is not affiliated to any organisation.

University of Pittsburgh

Organisation web address:

11. Review team members and their organisational affiliations.

Give the title, first name, last name and the organisational affiliations of each member of the review team. Affiliation refers to groups or organisations to which review team members belong.

Dr Andre L. Brown. University of Pittsburgh
Dr César G. Escobar-Viera. University of Pittsburgh
Dr Darren L Whitfield. University of Pittsburgh
Ms Ariel Shensa. University of Pittsburgh
Dr Jaime Sidani. University of Pittsburgh
Dr Brian A. Primack. University of Pittsburgh
Dr Michael P. Marshal. University of Pittsburgh

Dr Ron D. Stall. University of Pittsburgh
Mr Charles B. Wessel. University of Pittsburgh
Mr Cristian J. Chandler. University of Pittsburgh

12. * Funding sources/sponsors.

Give details of the individuals, organizations, groups or other legal entities who take responsibility for initiating, managing, sponsoring and/or financing the review. Include any unique identification numbers assigned to the review by the individuals or bodies listed.

The Fine Foundation

13. * Conflicts of interest.

List any conditions that could lead to actual or perceived undue influence on judgements concerning the main topic investigated in the review.

None

14. Collaborators.

Give the name and affiliation of any individuals or organisations who are working on the review but who are not listed as review team members.

15. * Review question.

State the question(s) to be addressed by the review, clearly and precisely. Review questions may be specific or broad. It may be appropriate to break very broad questions down into a series of related more specific questions. Questions may be framed or refined using PI(E)COS where relevant.

What is the impact of different patterns of social media use on depression among lesbian, gay, and bisexual populations?

16. * Searches.

Give details of the sources to be searched, search dates (from and to), and any restrictions (e.g. language or publication period). The full search strategy is not required, but may be supplied as a link or attachment.

We will execute literature searches in PubMed/MEDLINE (1946-Present), PsycINFO, Ovid® (1806 – Present) and SocINDEX, EBSCOhost (1895-Present). We will include quantitative and qualitative studies published in peer-reviewed journals, in English language, from 2003 (when MySpace, the first modern social media site started operations) until June 2017. We will allow manuscripts from conference proceedings only when full-research papers were required for submission and each submission went through complete peer rmeivnieowritiperso.cWesesd. eInficnleudSeMd Ustuadsieasnyhauvseagtoe fmoceuassuornemsoecniat l(em.ge.d, itaimues,efr(eSqMuUen) cayn,dmdoetpivraetsiosniotnoaumseo,negxspeexruieanIces while using, etc.). Depression comprised: major depressive disorder, bipolar depression, dysthymia, depressive symptoms, and psychologic distress. Sexual minorities are defined as lesbian, gay, bisexual, and men who have sex with men. Exclusion criteria includes: theses or dissertations, opinion pieces or reviews, gender minority population (e.g., transgender and gender non-binary), and use of short message services (not defined as social media).

17. URL to search strategy.

Give a link to the search strategy or an example of a search strategy for a specific database if available (including the keywords that will be used in the search strategies).

Alternatively, upload your search strategy to CRD in pdf format. Please note that by doing so you are consenting to the file being made publicly accessible.

Do not make this file publicly available until the review is complete

18. * Condition or domain being studied.

Give a short description of the disease, condition or healthcare domain being studied. This could include health and wellbeing outcomes.

Depression (major depressive disorder, bipolar depression, dysthymia, depressive symptoms, and psychologic distress)

19. * Participants/population.

Give summary criteria for the participants or populations being studied by the review. The preferred format includes details of both inclusion and exclusion criteria.

Lesbian, gay, and bisexual, individuals, and men who have sex with men

20. * Intervention(s), exposure(s).

Give full and clear descriptions or definitions of the nature of the interventions or the exposures to be reviewed.

Social media (SM) includes a variety of websites and mobile applications that enable users to create content and/or participate in online social networking (e.g., YouTube, Tumblr, Facebook, etc.). Social media use (SMU) is commonly defined as time (time elapsed while using SM over 24 hours), frequency (number of times people check their SM per day),number of platforms (sites or apps) used, closeness to online friends, and activities performed (e.g., posting updates, sharing pictures, etc.),as well as other SMU patterns (e.g., active vs passive use, negative experiences –such as experiencing cyberbullying–, problematic SMU, motivation to use SM, etc.).
We defined SMU as any usage measurement (e.g., time, frequency, motivation to use, experiences while using, etc.).

21. * Comparator(s)/control.

Where relevant, give details of the alternatives against which the main subject/topic of the review will be compared (e.g. another intervention or a non-exposed control group). The preferred format includes details of both inclusion and exclusion criteria.

Not applicable.

22. * Types of study to be included.

Give details of the types of study (study designs) eligible for inclusion in the review. If there are no restrictions on the types of study design eligible for inclusion, or certain study types are excluded, this should be stated. The preferred format includes details of both inclusion and exclusion criteria.

We will include quantitative and qualitative studies focusing on depression, social media use, and LGB populations, published in peer-reviewed journals, in English language, during or after 2003 (until June 2017). We will allow manuscripts from conference proceedings only when full-research papers were required for submission and each submission went through complete peer-review process. We will exclude theses or dissertations, opinion pieces, or reviews.

23. Context.

Give summary details of the setting and other relevant characteristics which help define the inclusion or exclusion criteria.

24. * Primary outcome(s).

Give the pre-specified primary (most important) outcomes of the review, including details of how the outcome is defined and measured and when these measurement are made, if these are part of the review inclusion criteria.

Our measure of depression includes major depressive disorder, bipolar depression, dysthymia,

depressive symptoms, and psychological distress.

Timing and effect measures

25. * Secondary outcome(s).

List the pre-specified secondary (additional) outcomes of the review, with a similar level of detail to that required for primary outcomes. Where there are no secondary outcomes please state ‘None’ or ‘Not applicable’ as appropriate to the review

None.

Timing and effect measures

26. Data extraction (selection and coding).

Give the procedure for selecting studies for the review and extracting data, including the number of researchers involved and how discrepancies will be resolved. List the data to be extracted.

Screening and data extraction will be completed using DistillerSR. We will upload structured forms into the software and use them throughout the entire data extraction process. Six researcher will independently screen all article titles and abstracts to generate a set of references for which there was any possibility for selection. Next, we will randomly assign three pairs of reviewers an equal number of references to assess full text of these studies to determine eligibility. We will calculate interrater reliability to ensure agreement among reviewers. To minimize the risk of reviewer bias, consensus meetings will be held between the first author and each pair of reviewers to resolve any differences, but only after independent screening of all articles.

Extraction forms will include six categories of information: (1) study logistics (e.g., setting, country, publication year, study design, funding source), (2) study population characteristics (including number of subjects, age, gender, race/ethnicity, sexual minorities included, education level, and income), (3) SMU (e.g. social networking sites, time use and frequency, scales, contextual measures), (4) health outcomes measured (including primary and secondary outcomes measured and scales), (5) main results and limitations, and (6) adequacy of reporting. To ensure accuracy, we will implement a quality control mechanism in which one reviewer completes a first data extraction and the second reviewer validates or disagrees with it. Again, disagreements will be resolved in consensus meetings with each pair and the first author.

27. * Risk of bias (quality) assessment.

State whether and how risk of bias will be assessed (including the number of researchers involved and how discrepancies will be resolved), how the quality of individual studies will be assessed, and whether and how this will influence the planned synthesis.

We will assess the reporting adequacy of all included studies. For quantitative studies, we will use the Strengthening the Reporting of Observational Studies in Epidemiology (STROBE) Statement Checklist v4.0. The STROBE statement consists of a checklist list of 22 items related to all sections of research manuscripts. STROBE provides reporting recommendations for studies that investigate associations between exposures and health outcomes. We will assign values of 0 – 2 to each check mark. Thus, total score for each manuscript could range from 0 – 22, in which 22 means the study fully met STROBE standards of adequate reporting.

For qualitative manuscripts, we will use the Consolidated Criteria for Reporting Qualitative Research (COREQ-32), a checklist of 32 items aimed at improving the quality of reporting individual interviews– and focus groups–generated data. We used the same previously explained mechanism to score each manuscript from 0 – 32, in which 32 means the study fully met COREQ-32 standards of adequate reporting. At least two reviewers will appraise each study and the first author will consult to resolve any disagreement.

28. * Strategy for data synthesis.

Give the planned general approach to synthesis, e.g. whether aggregate or individual participant data will be

used and whether a quantitative or narrative (descriptive) synthesis is planned. It is acceptable to state that a quantitative synthesis will be used if the included studies are sufficiently homogenous.

We will synthesize in table and narrative format the characteristics of studies on social media use and depression in samples which include men who have sex with men and lesbian, gay, and bisexual individuals. The characteristics that we will synthesize include the studies' author; country; year of publication; sample size, description, age range, and median age; race/ethnicity; female and sexual minorities composition of the sample; and reporting adequacy score. We will also synthesize in table and narrative format the studies' exposure assessment tool, outcome assessment tool, and main findings.

29. * Analysis of subgroups or subsets.

Give details of any plans for the separate presentation, exploration or analysis of different types of participants (e.g. by age, disease status, ethnicity, socioeconomic status, presence or absence or co-morbidities); different types of intervention (e.g. drug dose, presence or absence of particular components of intervention); different settings (e.g. country, acute or primary care sector, professional or family care); or different types of study (e.g. randomised or non-randomised).

None.

30. * Type and method of review.

Select the type of review and the review method from the lists below. Select the health area(s) of interest for your review.

Type of review

Cost effectiveness
No

Diagnostic
No

Epidemiologic
No

Individual patient data (IPD) meta-analysis
No

Intervention
No

Meta-analysis
No

Methodology
No

Network meta-analysis
No

Pre-clinical
No

Prevention
No

Prognostic
No

Prospective meta-analysis (PMA)
No

Qualitative synthesis
No

Review of reviews
No

Service delivery
No

Systematic review
Yes

Other
No

Health area of the review

Alcohol/substance misuse/abuse
No

Blood and immune system
No

Cancer
No

Cardiovascular
No

Care of the elderly
No

Child health
No

Complementary therapies
No

Crime and justice
No

Dental
No

Digestive system
No

Ear, nose and throat
No

Education
No

Endocrine and metabolic disorders
No

Eye disorders
No

General interest
No

Genetics
No

Health inequalities/health equity
No

Infections and infestations
No

International development
No

Mental health and behavioural conditions
Yes

Musculoskeletal
No

Neurological
No

Nursing
No

Obstetrics and gynaecology
No

Oral health
No

Palliative care
No

Perioperative care
No

Physiotherapy
No

Pregnancy and childbirth
No

Public health (including social determinants of health)
No

Rehabilitation
No

Respiratory disorders
No

Service delivery
No

Skin disorders
No

Social care
No

Surgery
No

Tropical Medicine
No

Urological
No

Wounds, injuries and accidents
No

Violence and abuse
No

31. Language.

Select each language individually to add it to the list below, use the bin icon to remove any added in error.
English

There is an English language summary.

32. Country.

Select the country in which the review is being carried out from the drop down list. For multi-national collaborations select all the countries involved.

United States of America

33. Other registration details.

Give the name of any organisation where the systematic review title or protocol is registered (such as with The Campbell Collaboration, or The Joanna Briggs Institute) together with any unique identification number assigned. (N.B. Registration details for Cochrane protocols will be automatically entered). If extracted data will be stored and made available through a repository such as the Systematic Review Data Repository (SRDR), details and a link should be included here. If none, leave blank.

34. Reference and/or URL for published protocol.

Give the citation and link for the published protocol, if there is one

Give the link to the published protocol.

Alternatively, upload your published protocol to CRD in pdf format. Please note that by doing so you are consenting to the file being made publicly accessible.

No I do not make this file publicly available until the review is complete

Please note that the information required in the PROSPERO registration form must be completed in full even if access to a protocol is given.

35. Dissemination plans.

Give brief details of plans for communicating essential messages from the review to the appropriate audiences.

We will publish the results of the systematic review in a peer-reviewed public health journal.

Do you intend to publish the review on completion?

Yes

36. Keywords.

Give words or phrases that best describe the review. Separate keywords with a semicolon or new line. Keywords will help users find the review in the Register (the words do not appear in the public record but are included in searches). Be as specific and precise as possible. Avoid acronyms and abbreviations unless these are in wide use.

Social media; Lesbian; Gay; Bisexual; Men who have Sex with Men; Depression; Cyberbullying

37. Details of any existing review of the same topic by the same authors.

Give details of earlier versions of the systematic review if an update of an existing review is being registered, including full bibliographic reference if possible.

38. * Current review status.

Review status should be updated when the review is completed and when it is published.
Please provide anticipated publication date

Review_Ongoing

39. Any additional information.

Provide any other information the review team feel is relevant to the registration of the review.

40. Details of final report/publication(s).

This field should be left empty until details of the completed review are available.

Give the link to the published review.
