# Supplementary material for: For Better or for Worse? A Systematic Review of the Evidence on Social Media Use and Depression Among Lesbian, Gay, and Bisexual Minorities
Source: JMIR Ment Health. 2018 Jul 23;5(3):e10496. doi: 10.2196/10496 (PMC6079300; doi:10.2196/10496)
Supplement: Multimedia Appendix 3 [file mental_v5i3e10496_app3.pdf]

Appendix 3. Online Search Strategy

A health sciences librarian guided the development of the search strategies. We used different combinations of terms for each of the three electronic databases (PubMed/MEDLINE; PsycINFO, Ovid; and SocINDEX, EBSCOhost) searched. The lists of search topics and terms used in electronic database are presented below.

PubMed/MEDLINE (1946-Present) Search Strategy

Search Domain: Lesbian, Gay, Bisexual, Transgender, Queer, and Intersex		
Search Strategy Step Number	Search Topic	Search Terms
1	LGBTQI	“Bisexuality”[Mesh] OR "Gender Dysphoria"[Mesh] OR "Gender Identity"[Mesh] OR "Homophobia"[Mesh] OR “Homosexuality”[Mesh] OR "Sexual and Gender Disorders"[Mesh] OR "Sexual Minorities"[Mesh] OR "Sex Reassignment Procedures"[Mesh] OR “Transgender Persons”[Mesh] OR "Transsexualism"[Mesh] OR affirmed gender[tiab] OR agender[tiab] OR androgynous[tiab] OR assigned sex[tiab] OR bicurious[tiab] OR biphobia[tiab] OR bisexual[tiab] OR bisexuality[tiab] OR bisexually[tiab] OR bisexuals[tiab] OR cisgender*[tiab] OR cross dresser*[tiab] OR cross gender*[tiab] OR cross sex*[tiab] OR crossgender[tiab] OR drag queen*[tiab] OR drag king*[tiab] OR F2M[tiab] OR female to male[tiab] OR GLB[tiab] OR GLBQ[tiab] OR GLBs[tiab] OR GLBT[tiab] OR GLBTQ[tiab] OR gay[tiab] OR gays[tiab] OR gender affirm*[tiab] OR gender change[tiab] OR gender creative[tiab] OR gender dysphori*[tiab] OR gender expansive[tiab] OR gender fluid[tiab] OR gender identit*[tiab] OR gender queer*[tiab] OR gender minorit*[tiab] OR gender non conform*[tiab] OR gender nonconform*[tiab] OR gender reassign*[tiab] OR gender spectrum[tiab] OR gender transform*[tiab] OR gender transition*[tiab] OR gender variant[tiab] OR genderqueer*[tiab] OR heteroflexible[tiab] OR homo sex*[tiab] OR homophob*[tiab] OR homosexual[tiab] OR homosexualities[tiab] OR homosexuality[tiab] OR homosexuals[tiab] OR intersex[tiab] OR lesbian[tiab] OR lesbianism[tiab] OR lesbians[tiab] OR lesbigay[tiab] OR LGB[tiab] OR LGBQ[tiab] OR LGBS[tiab] OR LGBT[tiab] OR LGBTI[tiab] OR LGBTQ[tiab] OR LGBTQI[tiab] OR LGBTT[tiab] OR M2F[tiab] OR male to female[tiab] OR men who have sex with men[tiab] OR msm[tiab] OR non heterosex*[tiab] OR

		nonheterosex*[tiab] OR pansex*[tiab] OR queer[tiab] OR queers[tiab] OR same gender loving[tiab] OR same sex attract*[tiab] OR same sex couple*[tiab] OR same sex relations*[tiab] OR sex change[tiab] OR sex reversal[tiab] OR sex transition[tiab] OR sexual identit*[tiab] OR sexual minorit*[tiab] OR sexual orientation[tiab] OR sexual preference[tiab] OR sexual racism[tiab] OR tgnc[tiab] OR third gender[tiab] OR trans female[tiab] OR trans gender*[tiab] OR trans health[tiab] OR trans individual*[tiab] OR trans male[tiab] OR trans men[tiab] OR trans people[tiab] OR trans persons[tiab] OR trans sexual*[tiab] OR trans woman[tiab] OR trans women[tiab] OR transfemale*[tiab] OR transgender*[tiab] OR transmale[tiab] OR transmales[tiab] OR transman[tiab] OR transmen[tiab] OR transmasculine[tiab] OR transpeople[tiab] OR transpersons[tiab] OR transphob*[tiab] OR transexual*[tiab] OR transsexual*[tiab] OR transvestit*[tiab] OR transwoman[tiab] OR transwomen[tiab] OR two spirit*[tiab] OR women loving women[tiab] OR women who have sex with women[tiab] OR YMSM[tiab]
Search Domain: Social Media		
Search Strategy Step Number	Search Topic	Search Terms
2A	Social Media	"Computer Communication Networks"[Mesh] OR "Internet"[Mesh] OR "Mobile Applications"[Mesh] OR "Online Systems"[Mesh] OR "Smartphone"[Mesh] OR "Social Media"[Mesh] OR "Social Networking"[Mesh] OR adam4adam[tiab] OR app[tiab] OR apps[tiab] OR computer mediated communicat*[tiab] OR cyber*[tiab] OR facebook*[tiab] OR flickr[tiab] OR geosocial network*[tiab] OR google plus[tiab] OR googleplus[tiab] OR grindr[tiab] OR grindr™[tiab] OR growlr[tiab] OR gsn[tiab] OR hook up [tiab] OR hook ups[tiab] OR hooking up[tiab] OR hookups[tiab] OR instagram*[tiab] OR instant messag*[tiab] OR internet daters[tiab] OR internet dating[tiab] OR iphone*[tiab] OR jack d[tiab] OR linkedin*[tiab] OR meet up[tiab] OR meetup[tiab] OR manhunt[tiab] OR men online[tiab] OR microblog*[tiab] OR mobile applicat*[tiab] OR mobile communicat*[tiab] OR mobile devic*[tiab] OR mobile phone*[tiab] OR mobile tech*[tiab] OR mobile telephone*[tiab] OR myspace[tiab] OR myspace™[tiab] OR networking sites[tiab] OR networking technol*[tiab] OR online commun*[tiab] OR online dating[tiab] OR online group*[tiab] OR online media[tiab] OR online sex[tiab] OR online social network*[tiab] OR online

		support[tiab] OR pinterest[tiab] OR professional network*[tiab] OR reddit*[tiab] OR scruff[tiab] OR sex online[tiab] OR sexting[tiab] OR smart phone*[tiab] OR smartphone*[tiab] OR snapchat[tiab] OR social media[tiab] OR social network*[tiab] OR text communicat*[tiab] OR text messag*[tiab] OR tinder[tiab] OR tumblr[tiab] OR tweet*[tiab] OR twitter*[tiab] OR virtual spac*[tiab] OR yelp[tiab] OR youtube[tiab] OR youtube™[tiab]
2B	Sexual Network/Partner/Seeking Sex on Internet/Online/Web/Websites	((sexual network[tiab] OR sexual networking[tiab] OR sexual networks[tiab] OR sexual partner*[tiab] OR ((seek[tiab] OR seeking[tiab]) AND sex[tiab]))) AND (internet[tiab] OR online[tiab] OR web[tiab] OR website*[tiab])
2C	Finding Partners on Internet/Online/Web/Websites	(find*[tiab] AND partner*[tiab]) AND (internet[tiab] OR online[tiab] OR web[tiab] OR website*[tiab])
2D	Sexual Behavior on Internet/Online/Web/Websites	"Sexual Behavior"[Mesh] AND (internet[title] OR online[title] OR web[title] OR website*[title])
2E	All Social Media Search Terms	Part 2A OR Part 2B OR Part 2C OR Part 2D
Search Domain: Depression Outcomes		
Search Strategy Step Number	Search Topic	Search Terms
3A	Depression	“Affective Symptoms"[Mesh] OR “Bipolar and Related Disorders”[Mesh] OR "Depression"[Mesh] OR "Mental Disorders"[Mesh:NoExp] OR "Mental Health"[Mesh] OR “Mood Disorders”[Mesh] OR “Self-Injurious Behavior”[Mesh] OR bipolar[tiab] OR cyclothym*[tiab] OR depress*[tiab] OR dysthymi*[tiab] OR hopeless*[tiab] OR mental health[tiab] OR mood[tiab] OR moods[tiab] OR psychological problem*[tiab] OR psychological distress*[tiab] OR psychological health[tiab] OR sadness[tiab] OR self esteem[tiab] OR self harm[tiab] OR self injur*[tiab] OR self mutilat*[tiab] OR suicid*[tiab]
3B	Related Depression Terms	"Adaptation, Psychological"[Mesh] OR “Bullying”[Mesh] OR "Loneliness"[Mesh] OR "Self Concept"[Mesh] OR "Social Stigma"[Mesh] OR “Stalking”[Mesh] OR "Stress, Psychological"[Mesh] OR bullying[tiab] OR loneliness [tiab] OR lonely[tiab] OR stigma[tiab] OR well being[tiab] OR wellbeing[tiab]
3C	All Depression Search Terms	Part 3A OR Part 3B
Search Domain: Lesbian, Gay, Bisexual, Transgender, Queer/Questioning, and Intersex, Social Media, and Depression Outcomes		
Search Strategy	Search Topic	Search Terms

Step Number		
4	LGBTQI AND Social Media AND Depression	Part 1 AND Part 2E AND Part 3C
Search Domain: Cyberbullying		
Search Strategy Step Number	Search Topic	Search Terms
5A	Cyberbullying	cyber bull*[tiab] OR cyber stalk*[tiab] OR cyber harass*[tiab] OR cyber victim*[tiab] OR cyberbull*[tiab] OR cyberharass*[tiab] OR cyberstalk*[tiab] OR cybervictim*[tiab]
5B	Internet Bullying/Harassment	(“Bullying”[Mesh] OR "Harassment, Non-Sexual"[Mesh:NoExp] OR "Sexual Harassment"[Mesh] OR "Stalking"[Mesh] OR bully*[tiab] OR harass*[tiab] OR victimizat*[tiab]) AND (“Internet”[Mesh] OR cyber*[tiab] OR internet[tiab] OR online[tiab] OR web[tiab] OR website*[tiab])
5C	All Cyberbullying Search Terms	Part 5A OR Part 5B
Search Domain: Lesbian, Gay, Bisexual, Transgender, Queer/Questioning, and Intersex and Cyberbullying		
Search Strategy Step Number	Search Topic	Search Terms
6	LGBTQI AND Cyberbullying	Part 1 AND Part 5C
Search Domain: All		
Search Strategy Step Number	Search Topic	Search Terms
7	LGBTQI AND Social Media AND Cyberbullying AND Depression	Part 4 OR Part 6

PsycINFO, Ovid® (1806 – Present) Search Strategy

Search Domain: Lesbian, Gay, Bisexual, Transgender, Queer, and Intersex		
Search Strategy Step Number	Search Topic	Search Terms
1A	American Psychological Association Thesaurus of Psychological Index Terms for LGBTQI	Androgyny/ or Bisexuality/ or Gender Equality/ or Gender Identity/ or exp Gender Identity Disorder/ or Hermaphroditism/ or Homosexual Parents/ or exp Homosexuality/ or "Homosexuality (attitudes toward)"/ or Same Sex Intercourse/ or Sex Change/ or Sex Roles/ or Sex Role Attitudes/ or Sociosexual Orientation/ or Transgender/ or "Transgender (attitudes toward)"/ or Transsexualism/ or Transvestism/
1B	Text Words (includes Title, Abstract, Table of Contents, Key Concepts) for LGBTQI	(agender or affirmed gender or androgyn* or assigned sex or bicurious or biphobia or bisexual* or cisgender* or cross dresser* or cross gender* or cross sex* or crossgender or drag queen* or drag king* or F2M or FTM or GBQ* or GLB or GLBQ or GLBs or GLBT or GLBTQ or gay or gays or gender affirm* or gender change or gender creative or gender disorder* or gender dysphor* or gender expansive or gender fluid or gender identit* or gender minorit* or gender non conform* or gender nonconform* or gender reassign* or gender spectrum or gender transform* or gender transition* or gender variant or genderqueer* or hermaphrodit* or heteroflexible or homo sex* or homo social or homophob* or homosex* or homosocial or intersex or lesbian* or lesbigay* or LGB* or M2F or MTF or MSM or MSMW or "men who have sex with men" or non heterosex* or nonheterosex* or pansex* or queer or queers or same gender lov* or same sex attract* or same sex couple* or same sex relations* or sex change or sex reversal or sex transition* or sexual identit* or sexual minorit* or sexual orientation or sexual preference or sexual racism or sex reassign* or tgnc or third gender or trans female* or trans health or trans gender* or trans individual* or trans male or trans men or trans people or trans persons or trans prejudice or trans sexual* or trans woman or trans women or transfemale* or transgender* or transmale or transmales or transman or transmen or transmasculin* or transpeople or transperson* or transphobi* or transexual* or transsexual* or transvestit* or transwoman or transwomen or two spirit* or WSMW or WSW or "women loving women" or "women who have sex with women" or YMSM).tw.
1C	LGBTQI Index Terms in Text	Part 1A OR Part 1B

	Words	
Search Domain: Social Media and Internet Sex Seeking		
Search Strategy Step Number	Search Topic	Search Terms
2A	American Psychological Association Thesaurus of Psychological Index Terms for Social Media	Computer Mediated Communication/ or Cyberbullying/ or exp Cybersex/ or Electronic Communication/ or Internet/ or Internet Addiction/ or Internet Usage/ or exp Mobile Devices/<br>or Online Community/ or Online Social Networks/ or Social Media/ or Websites/
2B	Text Words (includes Title, Abstract, Table of Contents, Key Concepts) for Sexual Network/Partner/Seeking Sex on Internet/Online/Web/Websites	(adam4adam or app or apps or computer mediated communicat* or cyber* or facebook* or geosocial network* or grindr or growlr or hook up* or hooked up or hooking up or hookup* or internet date* or internet dating or instant messag* or linkedin or men online or microblog* or mobile app* or mobile communicat* or mobile devic* or mobile phone* or mobile tech* or networking site or networking sites or online commun* or online dating or online group* or online media or online sex* or online social or online support or sex online or sexting or smart phone* or smartphone* or social media or text messag* or tweet* or twitter* or yik yak or youtube*).tw.
2C	Sexual Network/Partner/Seeking Sex	Psychosexual Behavior/ or (find* AND partner*).tw. or (sexual network* or sexual partner*).tw. or ((seek or seeking) and sex).tw.
2D	Internet/Online/Web/Websites	(internet or online or web or website*).tw.
2E	Sex Seeking on Internet	Part 2C AND Part 2D
2F	All Social Media and Internet Sex Seeking Search Terms	Part 2A OR Part 2B OR Part 2E
Search Domain: Depression Outcomes		
Search Strategy Step Number	Search Topic	Search Terms
3A	Depression	exp emotional adjustment/ or exp emotional states/ or exp emotions/ or exp major depression/ or mental health/ or exp self-destructive behavior/ or well being/ or depress*.mh. or (depress* or hopeless* or mental health OR mood or moods or self esteem or suicid*).tw. or (psychological adj (distress* or health or problem*)).tw.
3B	Related Depression Terms	exp bullying/ or coping behavior/ or exp harassment/ or personality traits/ or exp self-concept/ or

		exp stigma/ or exp stress/ or exp victimization/ or well being/ or (bully* or loneliness or lonely or stalking or stigma or well being or wellbeing).tw.
3C	All Depression Search Terms	Part 3A OR Part 3B
Search Domain: Lesbian, Gay, Bisexual, Transgender, Queer/Questioning, and Intersex, Social Media, and Depression Outcomes		
Search Strategy Step Number	Search Topic	Search Terms
4	LGBTQI AND Social Media AND Depression Outcomes	Part 1C AND Part 2F AND Part 3C
Search Domain: Cyberbullying		
Search Strategy Step Number	Search Topic	Search Terms
5A	Cyberbullying	(cyber bull* or cyber harass* or cyber stalk* or cyber victim* or cyberbull* or cyberharrass* or cyberstalk* or cybervictim*).tw.
5B	Bullying/Harassment	exp bullying/ or exp harassment/ or exp victimization or (bully* or harass* or stalk*).tw.
5C	All Previous Cyberbullying Search Terms	Part 2A OR Part 2B OR Part 2D
5D	All Bullying/Harassment Search Terms	Part 5B AND Part 5C
5E	All Cyberbullying Search Terms	Part 5A OR Part 5D
5F	LGBTQI AND Cyberbullying	Part 1C AND Part 5E
Search Domain: Lesbian, Gay, Bisexual, Transgender, Queer/Questioning, and Intersex, Social Media, Cyberbullying, and Depression Outcomes Combined with AND(Journal Articles Only)		
Search Strategy Step Number	Search Topic	Search Terms
6	LGBTQI AND Social Media AND Cyberbullying AND Depression	Part 4 OR Part 5F
Search Domain: Lesbian, Gay, Bisexual, Transgender, Queer/Questioning, and Intersex, Social Media, Cyberbullying, and Depression Outcomes Combined with AND (Journal Articles Only)		
Search Strategy Step Number	Search Topic	Search Terms
7	LGBTQI AND Social Media	Part 4 OR Part 5F (Journal Articles Only)

	AND Cyberbullying AND Depression	

SocINDEX, EBSCOhost (1895-Present) Search Strategy

Search Domain: Lesbian, Gay, Bisexual, Transgender, Queer, and Intersex		
Search Strategy Step Number	Search Topic	Search Terms
1	LGBTQI	agender or affirmed gender or androgyn* or assigned sex or bicurious or biphobia or bisexual* or cisgender* or cross dresser* or cross gender* or cross sex* or crossgender or drag queen* or drag king* or F2M or GBQ* or GLB or GLBQ or GLBs or GLBT or GLBTQ or gay or gays or gender affirm* or gender change or gender creative or gender disorder* or gender dysphor* or gender expansive or gender fluid or gender identit* or gender minorit* or gender non conform* or gender nonconform* or gender reassign* or gender spectrum or gender transform* or gender transition* or gender variant or genderqueer* or hermaphrodit* or heteroflexible or homo sex* or homo social or homophob* or homosex* or homosocial or intersex or lesbian* or lesbigay* or LGB* or M2F or MTF or MSM or MSMW or "men who have sex with men" or non heterosex* or nonheterosex* or pansex* or queer* or same gender lov* or same sex or sex change or sex reversal or sex transition* or sexual identit* or sexual minorit* or sexual orientation or sexual preference or sexual racism or sex reassign* or sociosexual orientat* or tgnc or third gender or trans female* or trans health or trans gender* or trans individual* or trans male or trans men or trans people or trans persons or trans prejudice or trans sexual* or trans woman or trans women or transfemale* or transgender* or transmale or transmales or transman or transmen or transmasculin* or transpeople or transperson* or transphobi* or transexual* or transsexual* or transvestit* or transwoman or transwomen or two spirit* or WSMW or WSW or "women loving women" or "women who have sex with women" or YMSM
Search Domain: Social Media and Internet Sex Seeking		
Search Strategy Step Number	Search Topic	Search Terms
2	Social Media	adam4adam or app or apps or bebo or “computer mediated communicat*” or crowdsourc* OR cyber* OR "dating site*" or facebook* or geosocial network* or grindr or growlr or “hook up*” or “hooked up*” or “hooking up” or hookup* or instant messag* or internet or "Jack’d" or linkedin or men online or microblog* or

		mobile app* or “mobile communicat*” or “mobile devic*” or “mobile phone*” or mobile tech* or myspace* or network site* or network website* or networking site* or networking website* or online commun* or online dating or online group* or “online identit*” or online media or online network* or online sex* or online social or online support or reddit or scruff or sex online or sexting or smart phone* or smartphone* or social media or text messag* or tweet* or twitter* or youtube*
Search Domain: Depression Outcomes		
Search Strategy Step Number	Search Topic	Search Terms
3	LGBTQI AND Social Media	Part 1 AND Part 2
Search Domain: Depression Outcomes		
Search Strategy Step Number	Search Topic	Search Terms
4	Depression	DE "Identity (Psychology)" or bully* or depress* or emotion* or harass* or happiness or loneliness or lonely or "mental health" or mood or moods or (psychological N5 (distress* or health or problem*) OR (self (destruct* or esteem or harm* or injur*) or stalk* or stigma or stress or suicid* or victimizat* or wellbeing or "well being"
Search Domain: LGBTQI, Social Media, and Depression		
Search Strategy Step Number	Search Topic	Search Terms
5	LGBTQI AND Social Media AND Depression	Part 3 AND Part 4
Search Domain: Cyberbullying		
Search Strategy Step Number	Search Topic	Search Terms
6	Cyberbullying	cyber bull* OR cyber stalk* OR cyber harass* OR cyber victim* OR cyberbull* OR cyberharass* OR cyberstalk* OR cybervictim*
Search Domain: Lesbian, Gay, Bisexual, Transgender, Queer, and Intersex and Cyberbullying		
Search Strategy Step Number	Search Topic	Search Terms
7	LGBTQI AND Cyberbullying	Part 1 AND Part 6
Search Domain: Lesbian, Gay, Bisexual, Transgender, Queer, and Intersex, Social Media, Cyberbullying, and Depression Outcomes		

Search Strategy Step Number	Search Topic	Search Terms
8	LGBTQI AND Social Media AND Cyberbullying AND Depression	Part 5 OR Part 7
Search Domain: Lesbian, Gay, Bisexual, Transgender, Queer, and Intersex, Social Media, Cyberbullying, and Depression Outcomes (Periodicals Only)		
Search Strategy Step Number	Search Topic	Search Terms
9	LGBTQI AND Social Media AND Cyberbullying AND Depression	Part 5 OR Part 7 (Periodicals Only)
