# Supplementary material for: For Better or for Worse? A Systematic Review of the Evidence on Social Media Use and Depression Among Lesbian, Gay, and Bisexual Minorities
Source: JMIR Ment Health. 2018 Jul 23;5(3):e10496. doi: 10.2196/10496 (PMC6079300; doi:10.2196/10496)
Supplement: Multimedia Appendix 4 [file mental_v5i3e10496_app4.pdf]

Appendix 4. Evaluation of Quantitative Studies Included using the Strengthening the Reporting of Observational Studies in Epidemiology (STROBE) Criteria

STROBE Reporting Criteria		*Morelli et al.<br>Italy, 2016<br>[54]*	*Gibbs & Rice<br>USA, 2016<br>[55]*	*Cenat et al.<br>Canada, 2015<br>[56]*	*Duong & Bradshaw<br>USA, 2014<br>[57]*	*Homan et al.<br>USA, 2014<br>[64]*	*Lester<br>USA, 2006<br>[58]*	*Cooper & Blumenfeld<br>USA, 2012<br>[59]*	*Ceglarek & Ward<br>USA, 2016<br>[60]*	*Ramsey et al.<br>USA, 2016<br>[61]*
Title and Abstract										
1	Clearly defined study design in title and summary abstract with findings	●	●	●	●	●		●	●	●
Introduction										
2	Study explains the background and rationale for the research	●	●	●	●	●		●	●	●
3	Study states specific objectives and any pre-specified hypotheses	●	●	●	●	●		●	●	●
Methods										
4	Study design elements are presented early in the paper	●	●	●	●	●		●	●	●
5	Study settings for recruitment, exposure, follow-up and data explained		●	●		●	●	●	●	●
6	Study gives the eligibility criteria and methods of participant selection		●	●	●	●	●	●	●	●
7	Study defines all outcomes, exposures, predictors, potential confounders and effect modifiers; provides diagnostic criteria if needed		●	●	●	●		●	●	●
8	Study explains measurement of variables in exposed and unexposed groups	●	●	●	●	●		●	●	●
9	Study explains any efforts to address potential bias		●	●	●	●				●
10	Study explains methods to arrive at the sample size		●	●	●	●		●	●	●
11	Study explains how variables were managed and any groupings created	●	●	●	●	●		●	●	●
12	Describes all statistical methods including (a) for controlling confounding, (b) examining subgroups, (c) explaining how missing data were handled, (d) described any way to account for sampling strategy, and (e) describe any sensitivity analyses	●	●	●	●	●			●	●
Results										
13	Study reports individuals at each stage of the study and gives reasons for non-participation at each stage		●					●	●	●
14	Study gives participant demographic, clinical and social characteristics while stating missing data for each variable		●						●	●
15	Study reports the number of outcome events or summary measures	●	●	●	●	●	●	●	●	●
16	Study reports (a) unadjusted estimates and, if applicable, confounder-adjusted estimates and their precision (e.g., 95% confidence interval); makes clear which confounders were		●	●				●	●	

	adjusted for and why they were included (b) report category boundaries when continuous variables were categorized and (c) if relevant, considers translating estimates of relative risk into absolute risk for a meaningful time period									
17	Study reports other analyses done—e.g., analyses of subgroups and interactions, and sensitivity analyses		●	●	●	●			●	
Discussion										
18	Study summarizes key results with reference to study objectives	●	●	●	●	●	●	●	●	●
19	Study discusses limitations of the study, taking into account sources of potential bias or imprecision; discusses both direction and magnitude of any potential bias	●	●	●	●	●			●	●
20	Study gives a cautious overall interpretation of results considering objectives, limitations, multiplicity of analyses, results from similar studies, and other relevant evidence	●	●	●	●	●			●	●
21	Study discusses the generalizability (external validity) of the study results		●	●	●	●	●		●	●
Other Information										
22	Study gives the source of funding and the role of the funders for the present study and, if applicable, for the original study on which the present article is based			●		●				
STROBE Total (of 22):		11	20	20	17	19	5	13	20	19

Note: STROBE criteria used from von Elm E, Altman DG, Egger M, et al. The Strengthening the Reporting of Observational Studies in Epidemiology (STROBE) Statement: Guidelines for Reporting Observational Studies. Ann Intern Med. 2007;147(8):573. doi:10.7326/0003-4819-147-8-200710160-00010.
