# Supplementary material for: For Better or for Worse? A Systematic Review of the Evidence on Social Media Use and Depression Among Lesbian, Gay, and Bisexual Minorities
Source: JMIR Ment Health. 2018 Jul 23;5(3):e10496. doi: 10.2196/10496 (PMC6079300; doi:10.2196/10496)
Supplement: Multimedia Appendix 5 [file mental_v5i3e10496_app5.pdf]

Appendix 5. Evaluation of Qualitative Studies Included using the Consolidated Criteria for Reporting Qualitative Research (COREQ-32)

COREQ Reporting Criteria	*Alang & Fomotar USA, 2014 [63]*	*Rubin & McClelland USA, 2015 [62]*
Domain 1: Research team and Reflexivity		
Study clearly states which authors conducted the interview/focus group	●	●
Credentials of the researchers are evident	●	
Researcher occupation at the time of the study is reported		
The gender of the researcher(s) is reported	●	●
The experience and training of the researcher is reported		
Study reports if a relationship with participants was established before study commencement		
Study reports what, if anything, participants knew about the researcher before the study commencement		
Study reports any interviewer characteristics such as bias or assumptions		●
Domain 2: Study Design		
Study states the methodological orientation and theory underpinning the study	●	●
Study explains the methods of participant selection	●	●
Study explains the method of participant approach	●	●
Study reports and explains the sample size included		●
Study explains reasons for participation refusal and attrition		
Study explains setting of data collection	●	●
Study reports any non-participants present during data collection		
Study reports demographic information about participants		●
If a guide was used, the researcher explains guide development and pilot testing	●	
If repeat interviews were given, the study reports the number and reason	●	
Study reports any audio/visual recording methods of data collection	●	●
Study reports if field notes were made during or after the focus group(s)		
Study reports the duration of focus group(s) and interview(s)	●	
Study reports if data saturation was reached		
Study reports if transcripts were reviewed by participants for comment or correction		
Domain 3: Analysis and Findings		
Study reports the number of data coders	●	
Researchers provided a description of the coding tree		
Researchers explained if themes were predetermined or derived from data	●	●
Researchers reported what software, if any, was used in data analysis		●
Researchers reported on the use of member checking		
Participant quotations, attributed to specific participants, were included to demonstrate themes	●	●
Study reports findings consistent with the data	●	●
Study presents clear themes	●	●
Study presents the diversity of cases and any minor themes determined from data	●	
COREQ Total Score:	17/32	15/32

Note: COREQ criteria extrapolated from Tong A, Sainsbury P, Craig J. Consolidated criteria for reporting qualitative research (COREQ): a 32- item checklist for interviews and focus group. *Int J Qual Heal Care*. 2007;19(6):349-357. doi:10.1093/intqhc/mzm042.
