# Supplementary material for: For Better or for Worse? A Systematic Review of the Evidence on Social Media Use and Depression Among Lesbian, Gay, and Bisexual Minorities
Source: JMIR Ment Health. 2018 Jul 23;5(3):e10496. doi: 10.2196/10496 (PMC6079300; doi:10.2196/10496)
Supplement: Multimedia Appendix 6 [file mental_v5i3e10496_app6.pdf]

TABLE 1. Characteristics of studies of social media use and depression including sexual minorities published between January 2003 and June 2017

Author(s), Country, Year	Design	Social Media Site / App	Participants							Appropriate Reporting Score[a]
			N	Sample Description	Age Range	Age Median	Race/Ethnicity (%)	Female (%)	Sexual Minorities (%)	
Morelli et al.<br>Italy, 2016<br>[54]	Cross-sectional survey	No specific site	1334	Mid- and high-school students and young adults	13 – 30	20.8	N/R	68	Lesbian/Gay<br>12.6	11[*]
Gibbs & Rice<br>USA, 2016<br>[55]	Cross-sectional survey	Grindr	195	Male users of a hook-up mobile application	18 – 24	22.2	Black 4.1<br>White 41.0<br>Latino 33.3<br>Asian 8.7<br>Mixed 11.8<br>Other 0.5	0	Gay<br>86<br>Bisexual<br>9.8	20[*]
Cenat et al.<br>Canada,<br>2015 [56]	Cross-sectional survey	No specific site	6540	Students from 34 participating high schools across Canada	14 – 20	15.4	N/R	56.3	Lesbian/Gay<br>1.3<br>Bisexual<br>10	20[*]
Rubin &<br>McClelland<br>USA, 2015<br>[62]	Individual interviews	Facebook	8	Female adolescent who reported being daily Facebook users	16 – 19	N/R	Black 37.5<br>White 0<br>Latino 37.5<br>Asian 25	100	Lesbian<br>62.5<br>Bisexual<br>37.5	15[#]
Duong &<br>Bradshaw<br>USA, 2014<br>[57]	Cross-sectional survey	No specific site	951	Sexual minority students, grades 9-12 from 105 NYC schools	N/R	N/R	Black 32.9<br>White 9.3<br>Latino 50.5<br>Asian 6.1	69.5	N/R	17[*]
Homan et al.<br>USA, 2014<br>[64]	Cross-sectional survey<br>Social network analysis	TrevorSpace	195	Users of a LGBQ social networking site	18 – 24	N/R	N/R	N/R	N/R	19[*]
Lester<br>USA, 2006<br>[58]	Cross-sectional survey	Bmezine	4700	Users of a body modification website	N/R	21	N/R	55	Lesbian/Gay<br>5<br>Bisexual	5[*]

									37.9	
Cooper & Blumenfeld USA, 2012 [59]	Cross-sectional survey	General blogs and discussion boards	310	National sample of middle and high school students who identified as LGB, or with same-sex attraction or LGBT allied youth	11 – 18	N/R	Black 2.9<br>White 69.4<br>Latino 7.4<br>Asian 3.2<br>Indigenous 0.6<br>Mixed 11.8<br>Other 3.5<br>Multi-racial 7.4	78.1	Lesbian/Gay/ Bisexual<br>80.6	13*
Alang & Fomotar USA, 2014 [63]	Netnography	Unidentified forum for new and expecting parents	N/R	Messages from an unidentified online forum for lesbian mothers with post-partum depression	N/R	N/R	N/R	100	Lesbian<br>100	17#
Ceglarek & Ward USA, 2016 [60]	Cross-sectional survey	No specific site	570	College students and community LGBTQ group members	18 – 24	20.2	Black 2.5<br>White 57.7<br>Latino 4.0<br>Asian 14.7<br>Indigenous 0.7<br>Multi-racial 3.0<br>Refused 2.3	62.6	Lesbian/Gay<br>6.8<br>Bisexual<br>5.4<br>Predominantly heterosexual<br>13.5	20*
Ramsey et al. USA, 2016 [61]	Cross-sectional survey	No specific site	634	Undergraduate college students	18 – 22	19.3	Black 5.8<br>White 79.0<br>Latino 6.2<br>Asian 3.2<br>Indigenous 0.7<br>Multi-racial 3.3<br>Other 0.8	66.8	Lesbian/Gay<br>7.6<br>Bisexual<br>4.4<br>Mostly Gay/ Lesbian<br>3.5<br>Mostly heterosexual<br>3.3<br>Questioning<br>0.8	19*

[a] Reporting adequacy was assessed using:
* the Strengthening the Reporting of Observational Studies in Epidemiology (STROBE, range 0 - 22) for quantitative studies, and
# the Consolidated Criteria for Reporting Qualitative Research 32 (COREQ-32, range 0 - 32) for qualitative studies.
N/R: Not reported
