# Supplementary material for: For Better or for Worse? A Systematic Review of the Evidence on Social Media Use and Depression Among Lesbian, Gay, and Bisexual Minorities
Source: JMIR Ment Health. 2018 Jul 23;5(3):e10496. doi: 10.2196/10496 (PMC6079300; doi:10.2196/10496)
Supplement: Multimedia Appendix 7 [file mental_v5i3e10496_app7.pdf]

TABLE 2. Exposure and Outcome Assessment and Main Findings of Studies of Social Media Use and Depression Including Sexual Minorities Published between January 2003 and June 2017

Author(s) Country, Year	Exposure Assessment Tool	Outcome Assessment Tool	Main Findings	Aspect of Minority Stress Theory Studied
Morelli et al. Italy, 2016 [54]	Modified version of the Sexting Behaviors Scale	12-item General Health Questionnaire	1097 (82.2%) reported sexting at least once. Males were more likely to be moderate (6.1%) and high (14.1%) users of sexting than females (2% and 4.1%; x2=60.96, p=0.000). Non-heterosexual participants had higher rates of using sexting behaviors than heterosexual participants (12.5% versus 6.5%; x2=8.39, p=.01). High and low sexting groups did not have significant difference in psychological distress.	Distal stressors
Gibbs & Rice USA, 2016 [55]	Sample was recruited exclusively from Grindr. However, overall use was not assessed	4-item Center for Epidemiological Studies Depression Scale (CES-D)	Moderate levels of depression symptoms (mean=7.1 on a scale 4-16, SD=3). Model including demographics, lifetime experience of homophobia, commitment to gay identity, social network composition, and enactment of identity commitment explains 29.5% of the variance in depression symptoms (F=3.2, p<0.01)	Distal stressors<br>Social support
Cenat et al. Canada, 2015 [56]	Item asking, "In the last 12 months, how many times someone has bullied you (rumors, intimidation, threatening, etc.) using the internet (Facebook, MySpace, MSN, email, text, etc.)	10-item Kessler Psychological Distress Scale<br><br>Item asking, "Have you ever seriously thought of committing suicide?"	Heterosexual and bisexual girls more likely than boys to report cyberbullying. Bisexual girls and boys more likely than heterosexuals to report cyberbullying (32.9% (CI 30-35.9) versus 21.4% (CI 20.0-22.7). 29.4% of gay and lesbian individuals reported homophobic bullying. Among sexual minority boys and girls, cyberbullying was significantly and independently associated with psychological distress, suicidal ideation, and low self-esteem. Compared to heterosexual boys, bisexual and lesbian girls were more likely to report psychological distress (β=1.45, p<0.001; β=0.74, p<0.05).	Distal stressors
Rubin & McClelland USA, 2015 [62]	Experience of being young, queer, and a person of color in an online network	Consequences of social exclusionary practices within an online network	All participants were daily Facebook users. Maintaining a Facebook profile is part of daily life, but also requires a lot of effort. Felt that Facebook profile was under constant surveillance by family and peers, so there was a need to monitor social interactions carefully, as they worried about being outed online. Individuals chose to remain "closeted" on Facebook, and spent a great deal of time ruminating about profile content. Participants described feelings of depression, shame, and	Distal stressors<br>Proximal stressors

			anxiety when monitoring Facebook content due to fears of social exclusion and unintentional “outing.”	
Duong & Bradshaw USA, 2014 [57]	Item from Youth Risk Behavior Survey (YRBS) asking "During the past 12 months, have you ever been electronically bullied, such as through e-mail, chat rooms, instant messaging, Web sites, or text messaging?"<br><br>Item asking, “In the past 12 months, have you ever been bullied on school property?”	Item asking, “During the past 12 months, how many times did you actually attempted suicide?”<br><br>Item asking, “During the past 12 months, how many times were you in a physical fight?”	Adjusted odd ratio of attempting suicide in the past 12 months was 3.07 (1.39-6.79) for youth who were cyberbullied only, 3.01 (1.09-8.33) school bullied only, and 5.10 (1.90-13.71) if bullied both cyber and school. Adjusted odds of engaging in one or more physical fight in the past 12 months was 4.34 (95% CI: 1.80-10.50) times greater among LGB youth who experienced both types of bullying compared to youth who were not bullied.	Distal stressors
Homan et al. USA, 2014 [64]	Social network structure graph	9-item Patient Healthcare Questionnaire (PHQ-9)	Social network analyses showed that participants with lower PHQ-9 scores were more tightly integrated into the social network than those with higher PHQ-9 scores ($p=0.05$), indicating that non-depressed individuals have more friends who know each other than depressed persons do.	Distal stressors<br>Social support
Lester USA, 2006 [58]	Sample was recruited exclusively from Bmezine. However, overall use was not assessed	Item asking, “How many times have you attempted suicide?”	Heterosexuals reported less suicidality than bisexual or homosexual group. Compared to heterosexual women, lesbians, bisexual, bisexual (heterosexual leaning), and bisexual (homosexual leaning) reported significantly higher rates of suicidality ($\chi=54.04$, $p<0.001$).	Distal stressors
Cooper & Blumenfeld USA, 2012 [59]	Item asking, “How often in an average week do you use communication technologies (e.g., blogging, chat rooms, discussion boards)<br>Item asking “how often in the last 30 days have you been harassed based on your sexual identity?”	Not provided	39% of LGBT and 32% of allied youth received angry, rude or vulgar messages at least 1-2 times per week, 8% of LGBT receiving messages 3 or more times a week; 56% of LGBT and 33% of allied participants felt depressed after being victims of cyberbullying; 35% of LGBT and 19% of allied youth had suicidal thoughts post being victims of cyberbullying.	Distal stressors

Alang & Fomotar USA, 2014 [63]	Assessment of the role of an online forum as source of social support	Experience of lesbian mothers with post-partum depression using a dedicated online forum	Sexual minority mothers may have a higher prevalence of Postpartum depression (PPD) than other men related to elevated risks of mental health problems in sexual minority women. Online forum served as a space where lesbian mothers with post-partum depression disclosed their experiences with the condition and fought feelings of loneliness and isolation. Forum was also used by these women to share successful ways of coping with post-partum depression. By participating in online groups, these lesbian mothers struggling with post-partum depression built a virtual community that provided sense of belonging, and emotional, instrumental, and informational support.	Social support
Ceglarek & Ward USA, 2016 [60]	Item asking, "How often do you use social networking sites?"<br><br>Item asking, "Which social networking sites do you use?"<br><br>Item asking, "How much these statements apply to you?" Example statement: "I use social networking sites to seek groups of people similar to myself"	26-item Brief Symptom Inventory	Sexual minority youth reported higher rates of motives for using social media than heterosexuals, as well as higher number social media sites used. Most frequent motives for social media use were social communication (M=3.40) and identity expression (2.57). Sexual minority reported overall higher levels of depression, anxiety, hostility, sensitivity, and loneliness than heterosexuals. Overall, social media use for identity exploration was significantly associated with greater reported depression symptoms (0.13) and loneliness (0.21), and greater levels of perceived social support were associated with fewer reported symptoms of depression and anxiety. Among sexual minorities, higher levels of perceived social support were associated with lower levels of loneliness (-0.027) and paranoia (-0.21); using social media to discuss issues around LGBT identity was negatively associated with anxiety (-0.35), hostility (-0.32) and paranoia (-0.43).	Social support
Ramsey et al. USA, 2016 [61]	Cyberbullying Victimization Scale of the Cyberbullying and Online Aggression Survey	Center for Epidemiological Studies Depression Scale-Revised (CESD-R)	Sexual minorities reported significantly higher levels of cyberbullying compared to heterosexuals. ANCOVA results showed that those with greater access to technology reported more cyberbullying $F(1,590)=4.84$, $p=0.028$ and this was more frequent among sexual minorities than heterosexuals $F(4,590)=6.44$, $p=0.000$. Sexual minorities reported increased levels of depression and recent cyberbullying or bullying at high school predicted current depression $F(8,570)=30.45$, $p=.000$.	Distal stressors
